# Editorial: Nurse fatigue: investigating burnout, health risks, and prevention strategies

**DOI:** 10.3389/fpubh.2026.1955137

**Published:** 2026-08-28

**Authors:** Ioannis Kouroutzis, Petros Galanis, Pavlos Sarafis, Maria Malliarou

**Affiliations:** 1 Healthcare Innovation Laboratory, Department of Nursing, University of Thessaly, Larissa, Greece; 2 Department of Nursing, National and Kapodistrian University of Athens, Athens, Greece

**Keywords:** burnout, nurse fatigue, nursing workforce, occupational health, patient safety, workforce sustainability

## Introduction

The sustainability of the global nursing workforce depends fundamentally on protecting nurses' physical, psychological, and occupational wellbeing. Against a background of workforce shortages, increasing care complexity, demographic change, and growing healthcare demands, nurse fatigue has emerged as one of the defining occupational health challenges facing contemporary healthcare systems. Far beyond physical exhaustion, fatigue is now recognized as a multidimensional phenomenon encompassing emotional depletion, impaired cognitive performance, sleep disruption, diminished recovery, and reduced professional wellbeing. Its consequences extend beyond individual health, influencing patient safety, quality of care, workforce retention, organizational performance, and the long-term sustainability of healthcare systems.

These considerations motivated the development of the Research Topic, *Nurse fatigue: investigating burnout, health risks, and prevention strategies*, with the aim of advancing understanding of the complex mechanisms underlying nurse fatigue and identifying evidence-informed strategies to prevent or mitigate its effects. We invited contributions examining burnout, turnover, emotional intelligence, occupational health risks, injury prevention, shift work, alarm fatigue, and interventions designed to improve nurses' health, wellbeing, and professional practice. The resulting Research Topic comprises 67 articles from researchers representing diverse healthcare systems, nursing specialties, and methodological traditions, providing a comprehensive overview of the contemporary evidence surrounding nurse fatigue.

The articles included in this Research Topic collectively demonstrate that nurse fatigue cannot be explained as the consequence of individual stress or inadequate resilience alone. Rather, they consistently illustrate that fatigue emerges through the interaction of organizational conditions, workplace demands, psychosocial environments, physical health, and individual psychological resources. Across the Research Topic, burnout was the most frequently investigated outcome, but the contributions also explored broader dimensions of occupational fatigue, including work engagement, professional identity, emotional intelligence, work–family balance, occupational injuries, sleep disturbance, work ability, alarm fatigue, workforce instability, and patient safety. By examining these interconnected dimensions, the Research Topic provides a more comprehensive understanding of fatigue as a systems-level phenomenon that affects both healthcare professionals and the organizations in which they work.

Despite differences in study design, population, and healthcare setting, the articles converge on several important observations. First, nurse fatigue is shaped by both organizational and individual determinants, emphasizing that prevention requires interventions targeting healthcare systems as well as healthcare professionals. Second, healthy work environments, supportive leadership, professional identity, resilience, emotional intelligence, and effective coping strategies consistently emerged as protective resources capable of mitigating the adverse effects of demanding clinical practice. Third, many of the included studies demonstrate that fatigue is closely linked to patient safety, workforce sustainability, and quality of care, reinforcing its importance as a strategic priority for healthcare organizations rather than solely an occupational health concern.

Rather than presenting fatigue as an inevitable consequence of nursing practice, the studies assembled in this Research Topic collectively argue that it is a preventable and modifiable challenge. Their findings support a shift from predominantly individual-focused approaches toward integrated strategies that combine organizational improvement, leadership, occupational health initiatives, professional development, and psychological support. In the sections that follow, we synthesize the principal themes emerging from this Research Topic and discuss their implications for nursing practice, leadership, education, policy, and future research.

## Burnout and occupational fatigue: understanding the multidimensional nature of nurse fatigue

Among the themes explored in this Research Topic, burnout emerged as the most consistently investigated manifestation of nurse fatigue. Although the included studies examined diverse nursing populations, healthcare settings, and organizational contexts, they consistently demonstrated that burnout is not merely an individual psychological response to demanding work but a multifaceted occupational phenomenon shaped by the interaction of organizational structures, workplace relationships, emotional demands, and individual resources. These findings reinforce the view that burnout should be regarded as a key indicator of workforce wellbeing and organizational health rather than simply an inevitable consequence of nursing practice.

Several contributions explored the organizational mechanisms underlying burnout. Studies by Li M. et al., Chen et al., Xu F. et al., and Bian et al. consistently showed that unfavorable work environments, effort–reward imbalance, work–family conflict, inadequate organizational support, and poor team climate contribute to burnout while simultaneously influencing professional competence, emergency preparedness, and patient safety. Rather than acting independently, these organizational factors appear to reinforce one another, creating working environments in which prolonged occupational demands exceed nurses' capacity for recovery and adaptation. Together, these studies highlight that interventions aimed at reducing burnout must extend beyond individual stress management and address the broader organizational conditions in which nursing care is delivered.

The Research Topic also provides important insights into the psychological processes linking occupational demands with burnout. Contributions by Chai et al., Gu et al., and Xia et al. demonstrated that resilience, social support, emotional regulation, self-worth, and professional identity influence nurses' ability to cope with occupational stress and reduce the adverse consequences of demanding clinical environments. Importantly, these studies do not suggest that resilience alone can prevent burnout. Instead, they indicate that psychological resources are most effective when developed within supportive organizational cultures that encourage professional growth, collaboration, and wellbeing.

Burnout was further examined as a mechanism connecting fatigue with broader workforce outcomes. Studies by Ji et al., Li J. et al., Zhou W. et al., Ma et al., and You et al. linked burnout to turnover intention, work withdrawal behaviors, change fatigue, and workforce instability, demonstrating that prolonged occupational strain influences not only nurses' health but also their commitment to the profession. These findings reinforce growing recognition that burnout has consequences extending well beyond the individual nurse, affecting staff retention, continuity of care, and the long-term resilience of healthcare organizations.

Overall, the studies included in this Research Topic reposition burnout from an individual outcome to a systems-level indicator of nursing work environments. They demonstrate that preventing burnout requires integrated strategies combining supportive leadership, healthy workplace cultures, equitable workloads, opportunities for recovery, and organizational policies that promote professional development and psychological wellbeing. This broader perspective provides an important conceptual foundation for understanding nurse fatigue as a multidimensional challenge with implications for workforce sustainability, patient safety, and healthcare quality.

## Organizational determinants of nurse fatigue: from working conditions to workforce sustainability

One of the clearest messages emerging from this Research Topic is that nurse fatigue is fundamentally shaped by the environments in which nurses work. Across healthcare settings and nursing specialties, the included studies consistently demonstrate that organizational factors—including leadership, staffing, work environment, organizational support, team functioning, and workplace culture—are not simply contextual influences but key determinants of nurses' health, professional performance, and workforce sustainability. This collective evidence shifts the focus from viewing fatigue as an individual occupational problem to recognizing it as an indicator of organizational health and the quality of nursing practice.

Several contributions examined how characteristics of the work environment influence nurses' wellbeing. Studies by Liu Y. et al. and Xu F. et al. demonstrated that supportive practice environments, positive team climate, and emotionally intelligent workplaces promote work engagement, reduce burnout, and strengthen safe nursing behaviors. These findings suggest that organizational cultures characterized by collaboration, trust, and effective communication not only improve nurses' professional experiences but also contribute directly to patient safety and the quality of care. Rather than reducing fatigue through isolated interventions, healthy work environments appear to enhance nurses' capacity to cope with occupational demands while maintaining professional effectiveness.

Leadership and organizational support also emerged as central protective factors throughout the Research Topic. Studies by Bian et al., Shi et al., and He et al. highlighted the importance of perceived organizational support, professional development, ethical leadership, and opportunities for participation in strengthening nurses' confidence, moral resilience, and engagement. Collectively, these studies indicate that supportive leadership extends beyond administrative management; it creates professional environments in which nurses feel valued, empowered, and capable of responding effectively to increasingly complex clinical demands. Such findings reinforce the critical role of nurse managers and healthcare leaders in fostering workplace cultures that support both staff wellbeing and high-quality patient care.

The articles further illustrate that organizational pressures extend beyond workload alone. Studies exploring work–life balance, work withdrawal, organizational change, and change fatigue demonstrated that demanding healthcare environments frequently affect nurses' professional commitment and personal wellbeing simultaneously. Contributions by Li J. et al., Zhou X. et al., Ma et al., You et al., and Hu et al. showed that repeated organizational pressures, insufficient recovery opportunities, and poorly managed workplace change contribute to disengagement, withdrawal behaviors, and diminished occupational wellbeing. These findings emphasize that sustaining a healthy nursing workforce requires healthcare organizations to consider not only staffing and workload but also the cumulative impact of organizational change on nurses' professional lives.

Another important contribution of this Research Topic is its attention to the psychosocial dimensions of the work environment. Klimanskaite et al. highlighted the value of salutogenic workplace characteristics that actively promote health and wellbeing, while Rachubińska et al. documented the psychosocial risks associated with rotating shift work. Other studies examining workplace violence, organizational silence, perceived deprivation, and interpersonal relationships further demonstrated that respectful communication, organizational justice, and psychological safety are essential components of healthy nursing workplaces. Together, these findings broaden conventional understandings of occupational health by recognizing that organizational culture is as important as physical working conditions in preventing fatigue and promoting workforce resilience.

Viewed collectively, the evidence presented in this Research Topic demonstrates that effective fatigue prevention cannot rely solely on strengthening individual resilience. Instead, sustainable improvements require organizational strategies that integrate supportive leadership, healthy work environments, equitable workloads, professional development, psychological safety, and meaningful staff participation in organizational decision-making. By positioning organizational health as a prerequisite for both nurse wellbeing and patient safety, this Research Topic provides compelling evidence that improving working conditions is not simply an employee welfare initiative but a strategic investment in healthcare quality, workforce sustainability, and resilient health systems.

Several contributions further highlight the importance of organizational culture in shaping nurses' professional experiences. Xu Z. et al. synthesized evidence on workplace violence against nurse managers, Liu X. et al. examined the impact of horizontal violence on burnout, Yang et al. examined organizational silencing and presenteeism among junior nurses, Fan et al. explored nurses' experiences with employee assistance programmes, Zhou X. et al. demonstrated the influence of authentic leadership and organizational climate on job embeddedness, while Seçkin and Çaglin examined the effects of leader-member exchange and perceived relative deprivation on job-related anxiety. Together, these studies reinforce the view that healthy work environments depend not only on staffing and workload but also on supportive leadership, organizational justice, effective communication, and psychologically safe workplace cultures.

## Psychological resources as protective mechanisms against nurse fatigue

While organizational conditions provide the context in which nurse fatigue develops, this Research Topic demonstrates that individual psychological resources play a crucial role in determining how nurses respond to occupational demands. Across the included studies, emotional intelligence, resilience, professional identity, self-efficacy, adaptive coping, work engagement, and moral resilience consistently emerged as factors associated with improved wellbeing and professional functioning. Importantly, however, the collective evidence does not support the view that these characteristics alone can prevent fatigue. Rather, the studies indicate that psychological resources are most effective when they are nurtured within supportive organizational environments that promote professional development, teamwork, and psychological safety.

Among the protective factors examined, emotional intelligence emerged as one of the most consistent mechanisms through which nurses maintain engagement and wellbeing. Studies by Liu Y. et al. demonstrated that emotional intelligence strengthened the positive relationship between supportive work environments and work engagement, while Gu et al. showed that emotional regulation, self-worth, and work engagement influence nurses' psychological responses to occupational stress. Together, these findings suggest that emotional competencies not only facilitate interpersonal relationships and communication but also enhance nurses' ability to adapt to demanding clinical environments without compromising their psychological wellbeing.

Resilience and professional identity were similarly identified as fundamental resources that promote adaptation to occupational challenges. Studies by Chai et al. demonstrated that resilience and social support attenuated the effects of moral distress on burnout among intensive care nurses, whereas Shi et al. highlighted the importance of moral resilience in supporting ethical behavior in clinical practice. Complementary findings from Xia et al. and Xie et al. further demonstrated that professional identity contributes to workforce stability and psychological wellbeing, suggesting that nurses who experience a stronger sense of professional purpose and belonging may be better equipped to manage occupational adversity while remaining committed to the profession.

The research findings also broaden understanding of nurses' active role in shaping their professional environments. Studies by Bian et al., Wang W. et al., and He et al. illustrate that self-efficacy, proactive job crafting, and nurses' voice behavior contribute to healthier workplaces by encouraging professional engagement and constructive responses to organizational challenges. Rather than portraying nurses as passive recipients of workplace conditions, these studies demonstrate that, when supported by responsive organizations, nurses actively influence both their own professional development and the functioning of their clinical environments.

Taken together, the evidence presented in this Research Topic highlights that psychological resources should be viewed as components of a broader organizational strategy to prevent nurse fatigue rather than as isolated individual attributes. Emotional intelligence, resilience, professional identity, self-efficacy, and adaptive coping strengthen nurses' capacity to respond to demanding clinical situations, but their benefits are maximized when healthcare organizations provide supportive leadership, opportunities for professional growth, healthy work environments, and cultures that value staff wellbeing. This integrated perspective represents one of the most important conceptual contributions of the Research Topic, demonstrating that sustainable workforce resilience depends on the continuous interaction between psychologically healthy professionals and healthy healthcare organizations.

The Research Topic also broadens understanding of the psychosocial factors that shape nurses' professional wellbeing. Beyond resilience and emotional intelligence, Liu S. et al. examined compassion fatigue and compassion satisfaction, Wang F. et al. explored the influence of coping styles and personality traits on occupational wellbeing, Li D. et al. investigated the roles of professional self-concept and social support, Wu J. et al. analyzed nurses' sense of professional achievement, while Wu F. et al. demonstrated the importance of medical narrative competence for retention intention. In addition, Fernandes et al. and Li L. et al. provided further evidence regarding emotional exhaustion and burnout across different nursing settings. Collectively, these studies illustrate that psychological wellbeing is influenced by a complex interaction of personal resources, professional identity, interpersonal relationships, and organizational support, emphasizing the multidimensional nature of nurse fatigue.

## Physical health, sleep, occupational risks, and patient safety

An important contribution of this Research Topic is its recognition that nurse fatigue is not solely a psychological phenomenon but a multidimensional occupational health challenge with consequences for physical wellbeing, clinical performance, and patient safety. Collectively, the articles demonstrate that prolonged exposure to demanding work environments affects multiple dimensions of nurses' health, extending from sleep disruption and impaired recovery to chronic physical conditions, reduced work ability, cognitive overload, and occupational injury. These findings reinforce the need to view fatigue through an integrated occupational health perspective rather than as an isolated symptom of workplace stress.

Sleep quality and recovery emerged as fundamental determinants of nurses' health throughout the Research Topic. Studies by Rachubińska et al., Qu et al., and Tan et al., demonstrated that rotating shifts, irregular work schedules, and prolonged occupational demands are associated with poorer physical and psychological health, highlighting the cumulative effects of disrupted recovery on nurses' wellbeing. Rather than representing temporary inconveniences, inadequate sleep and insufficient recovery appear to influence multiple aspects of professional functioning, reinforcing the importance of fatigue risk management, evidence-based scheduling practices, and organizational policies that promote adequate opportunities for rest.

The Research Topic further illustrates that the consequences of fatigue extend across multiple physiological systems. Studies by Peng et al. and other contributors examining somatic symptoms, migraine, musculoskeletal disorders, occupational skin health, and work ability demonstrate that sustained occupational demands are associated with a broad spectrum of health outcomes. These findings suggest that psychological and physical health cannot be considered independently, as chronic occupational strain affects nurses through interconnected biological, psychological, and behavioral pathways. This broader perspective supports comprehensive occupational health programmes that integrate physical health surveillance, ergonomic interventions, health promotion, and psychological support rather than addressing individual health problems in isolation.

Beyond its effects on individual wellbeing, the Research Topic consistently demonstrates that nurse fatigue has important implications for patient safety and healthcare quality. Studies by Xu F. et al. and Bian et al. showed that supportive organizational environments, reduced burnout, and stronger professional competence contribute to safer nursing practice and greater emergency preparedness. These findings reinforce the increasingly recognized relationship between workforce wellbeing and patient outcomes, suggesting that protecting nurses from excessive fatigue is also a strategy for strengthening clinical performance, reducing safety risks, and improving healthcare quality.

A distinctive strength of this Research Topic is the attention given to alarm fatigue, an emerging challenge in increasingly technology-intensive healthcare environments. The review by Klimanskaite et al. highlights how excessive alarm exposure, false alarms, high cognitive workload, inadequate training, and organizational factors contribute to reduced vigilance and delayed responses to clinically significant alarms. Importantly, the review demonstrates that effective alarm management depends not only on technological improvements but also on education, standardized protocols, interdisciplinary collaboration, and organizational commitment to reducing unnecessary cognitive burden. This perspective illustrates how technological innovation, while improving patient monitoring, may inadvertently contribute to occupational fatigue if implementation is not accompanied by appropriate system-level support.

Taken together, the evidence presented in this Research Topic demonstrates that fatigue should be understood as a multidimensional occupational health challenge that simultaneously affects nurses' physical health, psychological wellbeing, cognitive functioning, and clinical performance. Sleep disturbance, impaired recovery, chronic health conditions, occupational injury, cognitive overload, and patient safety are therefore not isolated outcomes but interconnected manifestations of prolonged occupational strain. These findings support the development of comprehensive fatigue-prevention strategies that combine healthy scheduling practices, occupational health services, ergonomic interventions, effective alarm governance, and organizational policies that prioritize both workforce wellbeing and safe patient care. By integrating occupational health with patient safety, this Research Topic broadens current understanding of nurse fatigue and provides a strong foundation for future interventions designed to improve both nurses' health and healthcare quality.

An additional strength of this Research Topic is its broader characterization of occupational health across diverse nursing specialties and professional roles. Studies by Qalawa et al., Li N. et al., and Jarosz and Młynarska further illustrated the heterogeneous clinical manifestations of occupational strain, including migraine, somatization symptoms, and specialty-specific fatigue. Complementing these findings, Yuan et al., Wang N. et al., and Wang Y. et al. emphasized the importance of mental workload, work ability, and occupational stress as key determinants of professional performance and wellbeing. In addition, Luo et al., Zeng et al., and Wu J. et al. expanded understanding of occupational risks by examining exposure hazards, occupational fatigue in specialized nursing settings, and fatigue among nursing managers. Collectively, these studies demonstrate that occupational health should be understood through a comprehensive perspective that integrates physical health, cognitive workload, occupational risks, and organizational support across diverse nursing contexts.

## From nurse fatigue to workforce sustainability: implications for practice, leadership, and policy

Beyond advancing knowledge of the determinants and consequences of nurse fatigue, this Research Topic highlights a broader challenge confronting healthcare systems worldwide: sustaining a healthy, engaged, and resilient nursing workforce. Although the articles address diverse clinical settings, professional groups, and research questions, they consistently demonstrate that fatigue is closely linked to workforce sustainability through its effects on professional engagement, work ability, retention, organizational commitment, and the quality and safety of patient care. The evidence suggests that addressing nurse fatigue is not simply an occupational health priority but a strategic imperative for ensuring resilient healthcare systems.

Several contributions illustrate how fatigue influences nurses' decisions to remain in or leave the profession. Studies by Ji et al., Xia et al., Li J. et al., Zhou W. et al., Ma et al., and You et al. consistently demonstrate that burnout, work–life imbalance, organizational stress, and change fatigue contribute to work withdrawal behaviors, turnover intention, and workforce instability. Rather than representing isolated workforce outcomes, these findings indicate that retention is closely associated with nurses' everyday experiences of organizational support, professional recognition, and opportunities for recovery. Consequently, strategies aimed at strengthening workforce sustainability should focus not only on recruitment but also on creating working environments in which nurses are able to develop, remain engaged, and build long-term professional careers.

The Research Topic also underscores the importance of strengthening nurses' participation in organizational life. Studies exploring job crafting, voice behavior, organizational silence, and narrative competence suggest that nurses contribute actively to the development of healthier workplaces when they are encouraged to participate in decision-making, innovation, and professional dialogue. These findings reinforce contemporary models of nursing leadership that emphasize collaboration, empowerment, and shared responsibility for improving both working conditions and patient care. Supporting nurses as active partners in organizational development may therefore represent an important strategy for reducing fatigue while simultaneously strengthening professional engagement and organizational resilience.

Perhaps the most important contribution of this Research Topic is its consistent demonstration that sustainable solutions require integrated, multilevel approaches. Throughout the Research Topic, interventions targeting individual resilience, emotional intelligence, coping strategies, and psychological wellbeing are shown to be valuable but insufficient when implemented in isolation. Their effectiveness depends on organizational environments characterized by supportive leadership, appropriate staffing, healthy work cultures, effective communication, opportunities for recovery, and policies that recognize nurses' wellbeing as an essential component of healthcare quality. This integrated perspective represents a significant conceptual shift, moving the discussion beyond individual adaptation toward shared organizational and system-level responsibility for preventing fatigue.

The diversity of methodological approaches represented in the Research Topic further strengthens these conclusions. In addition to cross-sectional studies, the Research Topic includes multicenter investigations, latent profile analyses, mediation and moderation models, predictive modeling, qualitative research, systematic reviews, and bibliometric analyses. Together, these complementary approaches provide a richer understanding of the complex relationships linking organizational factors, psychological resources, physical health, and professional outcomes. At the same time, the predominance of observational designs highlights the need for future longitudinal, intervention, and implementation studies capable of evaluating the effectiveness of fatigue-prevention strategies across different healthcare systems and cultural contexts.

The evidence presented in this Research Topic supports a fundamental shift in how nurse fatigue should be understood and addressed. Fatigue should no longer be viewed solely as an unavoidable consequence of demanding clinical work or as an issue to be managed primarily by individual nurses. Instead, it should be recognized as a measurable indicator of organizational health, workforce sustainability, and healthcare quality. Addressing nurse fatigue therefore requires coordinated action across clinical practice, leadership, education, occupational health, and public policy. By positioning nurses' wellbeing as a strategic investment rather than an operational challenge, the studies in this Research Topic provide a strong foundation for developing healthier workplaces capable of sustaining both the nursing workforce and the delivery of safe, high-quality patient care.

The diversity of nursing populations represented in this Research Topic further illustrates that fatigue prevention should be adapted to different professional roles and clinical contexts. Dang et al. classified nursing roles in critical infectious disease care, Mathebula et al. explored the psychosocial wellbeing of nurse educators, Xu J. et al. mapped emerging trends in research on newly graduated nurses, Wu J. et al. investigated occupational fatigue among nursing managers, Zhang et al. synthesized evidence regarding occupational health risks in operating room personnel, and Kagan et al. examined burnout within the allergy nursing workforce in the United States. Collectively, these studies demonstrate that occupational challenges differ across specialties, career stages, and healthcare environments, supporting the development of context-specific interventions while maintaining common organizational commitments to workforce wellbeing and sustainability.

## Concluding remarks

The studies assembled in this Research Topic provide a comprehensive and contemporary perspective on one of the most significant challenges facing the nursing profession worldwide. Although they address diverse healthcare settings, nursing specialties, and research questions, they converge on a common message: nurse fatigue is a complex, multidimensional phenomenon that cannot be adequately understood through an individual or symptom-based perspective alone. Instead, the collective evidence demonstrates that fatigue emerges from the continuous interaction between organizational structures, workplace demands, psychological resources, physical health, and professional practice. This integrated understanding represents one of the principal contributions of the Research Topic and provides a stronger conceptual foundation for future research and intervention.

Importantly, the Research Topic moves beyond the traditional focus on burnout as the sole indicator of occupational strain. The included articles demonstrate that fatigue influences multiple dimensions of nurses' professional and personal lives, including work engagement, professional identity, emotional intelligence, resilience, sleep quality, occupational health, work ability, workforce retention, and patient safety. By bringing together these complementary perspectives, the Research Topic illustrates that preventing fatigue requires coordinated actions that extend across clinical practice, leadership, education, occupational health, workforce planning, and healthcare policy. Such an integrated approach acknowledges that sustainable improvements in nurses' wellbeing cannot be achieved through individual interventions alone but require organizational and system-level commitment to creating healthier work environments.

Another important strength of this Research Topic lies in its methodological diversity. The combination of observational studies, multicenter investigations, latent profile analyses, mediation models, predictive modeling, qualitative research, systematic reviews, and bibliometric analyses reflects the growing maturity of research on nurse fatigue and broadens current understanding of its determinants and consequences. At the same time, the predominance of cross-sectional research highlights the continuing need for longitudinal, intervention, and implementation studies capable of evaluating fatigue-prevention strategies and translating evidence into sustainable improvements in nursing practice.

The Research Topic also expands current understanding of fatigue by addressing emerging occupational health challenges that have received comparatively limited attention in nursing research. Studies by Bin et al. and Jiang et al. further highlighted the importance of sleep disturbance, night-shift workload, and their psychological consequences, while Ni et al. examined the mental health impact of epidemic conditions on healthcare professionals. In addition, Zhu et al. provided valuable qualitative insights into the mechanisms underlying alarm fatigue, complementing quantitative evidence presented elsewhere in the Research Topic. Together, these studies illustrate the growing diversity of methodological approaches and research priorities that continue to broaden the conceptualization of nurse fatigue and occupational wellbeing.

Ultimately, the evidence presented throughout this Research Topic supports a fundamental shift in how nurse fatigue should be viewed within healthcare systems. Fatigue should no longer be regarded as an inevitable consequence of nursing or as a problem that individual nurses are expected to manage independently. Rather, it should be recognized as a measurable indicator of organizational health, workforce sustainability, and healthcare quality. This perspective places responsibility not only on individual professionals but also on healthcare organizations, educational institutions, professional bodies, and policy-makers to create environments that enable nurses to deliver safe, effective, and compassionate care while maintaining their own health and wellbeing.

We hope that the studies presented in this Research Topic will encourage continued interdisciplinary collaboration and stimulate the development of innovative, evidence-informed strategies to prevent nurse fatigue across diverse healthcare settings. More importantly, we hope that the collective evidence assembled here contributes to a broader cultural shift in which protecting nurses' wellbeing is recognized not simply as an occupational health objective, but as a prerequisite for resilient healthcare systems, high-quality patient care, and the future sustainability of the nursing profession.

